# The hierarchical organization of the precuneus captured by functional gradients

**DOI:** 10.1007/s00429-023-02672-5

**Published:** 2023-06-28

**Authors:** Ping Jiang, Shunshun Cui, Shanwen Yao, Huanhuan Cai, Jiajia Zhu, Yongqiang Yu

**Affiliations:** 1 Department of Radiology, The First Affiliated Hospital of Anhui Medical University, Hefei, 230022 China; 2 Research Center of Clinical Medical Imaging, Anhui Province Hefei, 230032 China; 3 Anhui Provincial Institute of Translational Medicine, Hefei, 230032 China

**Keywords:** Precuneus, Functional gradients, Hierarchical organization, Functional MRI, Resting-state functional connectivity

## Abstract

The precuneus shows considerable heterogeneity in multiple dimensions including anatomy, function, and involvement in brain disorders. Leveraging the state-of-the-art functional gradient approach, we aimed to investigate the hierarchical organization of the precuneus, which may hold promise for a unified understanding of precuneus heterogeneity. Resting-state functional MRI data from 793 healthy individuals were used to discover and validate functional gradients of the precuneus, which were calculated based on the voxel-wise precuneus-to-cerebrum functional connectivity patterns. Then, we further explored the potential relationships of the precuneus functional gradients with cortical morphology, intrinsic geometry, canonical functional networks, and behavioral domains. We found that the precuneus principal and secondary gradients showed dorsoanterior-ventral and ventroposterior-dorsal organizations, respectively. Concurrently, the principal gradient was associated with cortical morphology, and both the principal and secondary gradients showed geometric distance dependence. Importantly, precuneus functional subdivisions corresponding to canonical functional networks (behavioral domains) were distributed along both gradients in a hierarchical manner, i.e., from the sensorimotor network (somatic movement and sensation) at one extreme to the default mode network (abstract cognitive functions) at the other extreme for the principal gradient and from the visual network (vision) at one end to the dorsal attention network (top-down control of attention) at the other end for the secondary gradient. These findings suggest that the precuneus functional gradients may provide mechanistic insights into the multifaceted nature of precuneus heterogeneity.

**Supplementary Information:**

The online version contains supplementary material available at 10.1007/s00429-023-02672-5.

## Introduction

The precuneus (PCun), localized to the posterior medial portion of the parietal cortex, is an anatomically and functionally heterogeneous brain structure (Cavanna and Trimble 2006; Luo et al. 2020; Zhang et al. 2014). Through its widespread connections with both cortical and subcortical regions, the PCun has played a pivotal role in neural communication and coordination in multiple large-scale brain networks (e.g., the default mode and frontoparietal networks) (Dorfel et al. 2009; Utevsky et al. 2014; Yang et al. 2014; Cunningham et al. 2017), such that it is generally assumed to subserve a rich range of high-level cognitive functions (Cavanna and Trimble 2006; Vanlierde et al. 2003; Lundstrom et al. 2005; Haj et al. 2014; Li et al. 2015; Al-Ramadhani et al. 2021). From an evolutionary perspective, the study of the PCun is also of vital importance (Cavanna and Trimble 2006; Margulies et al. 2009; Zhang et al. 2017) as empirical evidence suggests that PCun expansion is a key feature of modern human evolution and a major source of human cognitive specializations (Bruner et al. 2017). Furthermore, clinical neuroimaging research has documented that PCun abnormalities are critically involved in the neuropathology of many neurological and psychiatric disorders (Zhu et al. 2018; Kitamura et al. 2021; Gonen et al. 2020; Dong et al. 2020b; Frings et al. 2010), but with the exact location and nature of abnormalities varying across diseases. Despite these findings in basic and clinical neuroscience, a potentially mechanistic framework for a unified understanding of the various facets of PCun heterogeneity remains to be established.

There is diverse and convergent evidence for the existence of hierarchical gradients in multiscale brain organization, which is reflected in structure, function, connectivity, and gene expression (Margulies et al. 2016; Bajada et al. 2017; Wagstyl et al. 2015; Paquola et al. 2020, 2019; Huntenburg et al. 2018; Shine et al. 2019; Vogel et al. 2020; Gomez et al. 2019). Dimensionality reduction techniques (e.g., diffusion embedding algorithm) have been widely applied to high-dimensional resting-state functional connectivity (rsFC) data from resting-state functional magnetic resonance imaging (rs-fMRI) to characterize the hierarchical organization of the brain. This analytic procedure would yield a parsimonious set of principal components describing smooth transitions of rsFC patterns across brain areas, referred to as functional gradients (Hong et al. 2020; Bajada et al. 2020; Vos de Wael et al. 2020). Taking advantage of functional gradients, emerging efforts have recapitulated meaningful organizational principles for multiple brain structures such as the cerebral cortex (Margulies et al. 2016), cerebellum (Guell et al. 2018), primary somatosensory cortex (Ngo et al. 2021), striatum (Marquand et al. 2017), insula (Tian and Zalesky 2018; Wang et al. 2023), hippocampus (Vos de Wael et al. 2018; Bayrak et al. 2022), thalamus (Yang et al. 2020) and angular gyrus (Song et al. 2023), making functional gradients recently gain increasing attention in the neuroimaging and network neuroscience community. For example, Margulies et al. (2016) described a dominant sensorimotor-to-transmodal gradient in the cerebral cortex, in favor of the well-defined central principle that macroscale anatomy reflects a functional hierarchy from primary to transmodal processing. Moreover, some functional gradients have shown underlying structural basis, geometric distance dependence, correspondence with canonical functional networks, and involvement in specific behavioral domains (Zhu et al. 2018; Ngo et al. 2021; Yang et al. 2020). Despite the broad utility, there is a paucity of literature leveraging the functional gradient approach to investigate the hierarchical organization of the PCun, clarification of which might provide a mechanistic account for PCun heterogeneity.

**Fig. 1:**
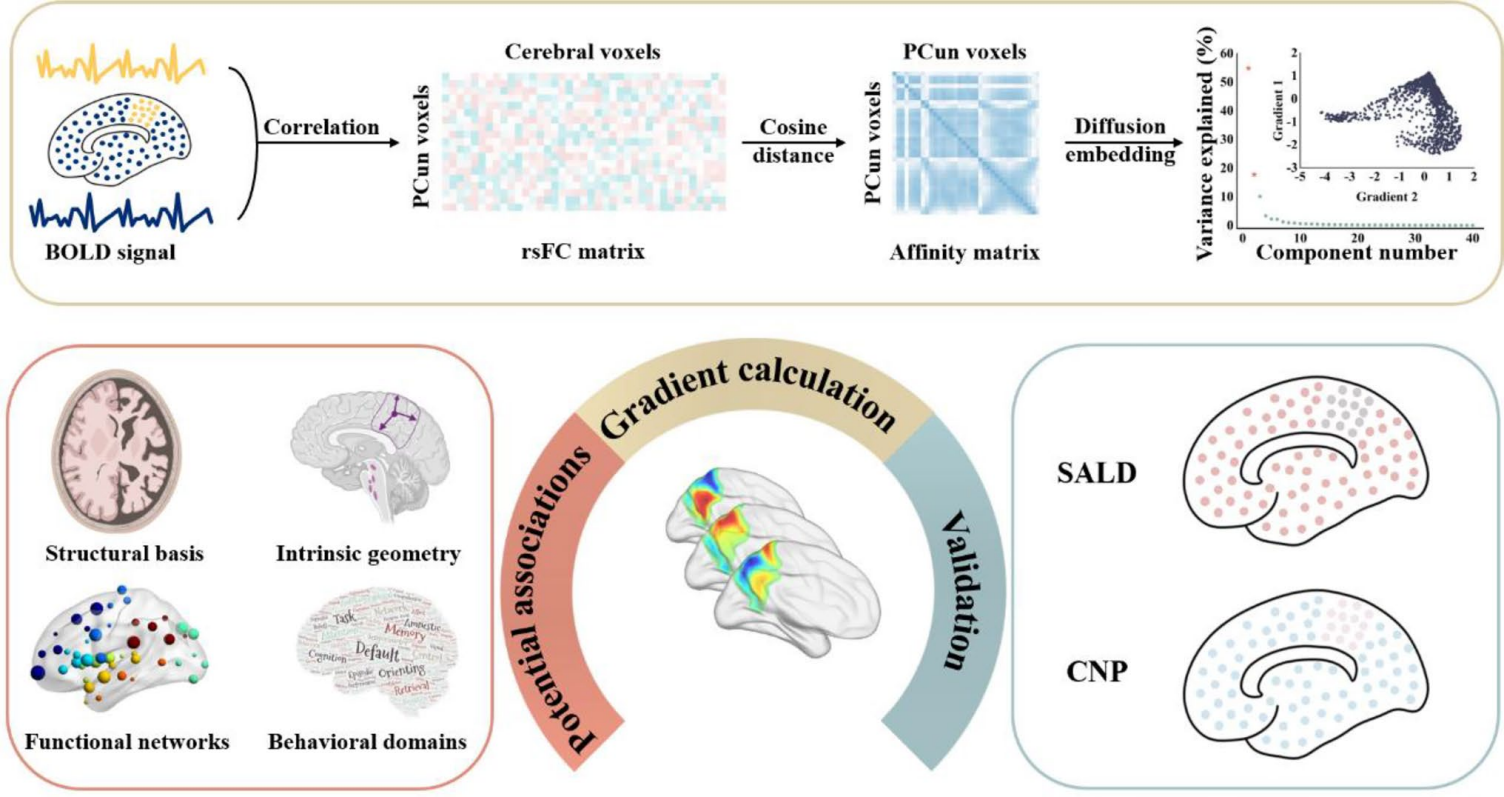
Summary of the analysis pipeline. rs-fMRI data were obtained from a discovery dataset and two independent cross-race, cross-scanner validation datasets (SALD and CNP). Functional gradients of the PCun were calculated based on the voxel-wise PCun-to-cerebrum rsFC patterns using diffusion embedding (top panel) and validated in the SALD and CNP datasets (right panel). For the resultant functional gradients, we further explored their potential relationships with cortical morphology, intrinsic geometry, canonical functional networks, and behavioral domains (left panel). *BOLD* blood-oxygen-level-dependent, *PCun* precuneus, *rsFC* resting-state functional connectivity, *SALD* Southwest University Adult Lifespan Dataset, *CNP* Consortium for Neuropsychiatric Phenomics, *rs-fMRI* resting-state functional magnetic resonance imaging

To address this missing gap, we used rs-fMRI data from 793 healthy individuals (361 from our discovery dataset, 329 from Southwest University Adult Lifespan Dataset, and 103 from Consortium for Neuropsychiatric Phenomics) to discover and validate functional gradients of the PCun, which were calculated based on the voxel-wise PCun-to-cerebrum rsFC patterns. Then, we further explored the potential relationships of PCun functional gradients with cortical morphology, intrinsic geometry, canonical functional networks, and behavioral domains. A schematic overview of the analysis pipeline is shown in Fig. 1.

## Methods

### Participants

Our study included a discovery dataset along with two independent cross-scanner and cross-race validation datasets. The discovery participants were healthy adults of Chinese Han and right-handedness, recruited from the local universities and community through poster advertisements. Exclusion criteria included neuropsychiatric or severe somatic disorder, a history of head injury with loss of consciousness, pregnancy, MRI contraindications, and a family history of psychiatric illness among first-degree relatives. This study was approved by the ethics committee of The First Affiliated Hospital of Anhui Medical University. Written informed consent was obtained from all participants after they had been given a complete description of the study. The validation samples were from two publically available datasets: Southwest University Adult Lifespan Dataset (SALD) (Wei et al. 2018) and Consortium for Neuropsychiatric Phenomics (CNP) (Poldrack et al. 2016). Of note, we solely selected the healthy adults from the cross-disorder CNP dataset. Full details regarding the two validation samples (e.g., ethics, informed consent, inclusion and exclusion criteria, among others) have been described in the data descriptor publications (Wei et al. 2018; Poldrack et al. 2016). To rule out the potential influence of neurodevelopment and neurodegeneration, all the participants were restricted to an age range of 18–60 years. Additionally, participants with poor image quality or excessive head motion during scanning were excluded. This brought the final samples used in this study to 361 in the discovery dataset, 329 in the SALD dataset, and 103 in the CNP dataset. Details of the demographic data of the three datasets are presented in Table S1 in the Supplementary materials.

### Image acquisition

MRI data of the discovery sample were obtained using the 3.0-Tesla General Electric Discovery MR750w scanner, and those of the validation samples were acquired using the 3.0-Tesla Siemens Trio scanners. Details of the resting-state fMRI protocols for the three datasets can be found in Table S2 in the Supplementary materials.

### fMRI data preprocessing

Resting-state blood-oxygen-level-dependent (BOLD) data were preprocessed using Statistical Parametric Mapping software (SPM12, http://www.fil.ion.ucl.ac.uk/spm) and Data Processing & Analysis for Brain Imaging (DPABI, http://rfmri.org/dpabi) (Yan et al. 2016). The first several time points (discovery: 10, SALD: 10, CNP: 5) for each participant were discarded to allow the signal to reach equilibrium and the participants to adapt to the scanning noise. The remaining volumes were corrected for the acquisition time delay between slices. Then, realignment was performed to correct the motion between time points. Head motion parameters were assessed by calculating the translation in each direction and the angular rotation on each axis for each volume. All BOLD data of the final sample were within the defined motion thresholds (i.e., maximum translation or rotation < 2 mm or 2^°^). We also computed frame-wise displacement (FD), which measures the volume-to-volume changes in head position. Several nuisance covariates (the linear drift, the estimated motion parameters based on the Friston-24 model, the spike volumes with FD > 0.5 mm, the white matter signal, and the cerebrospinal fluid signal) were regressed out from the data. The datasets were then band-pass filtered using a frequency range of 0.01 to 0.1 Hz. In the normalization step, individual structural images were firstly co-registered with the average functional images; then the transformed structural images were segmented and normalized to the Montreal Neurological Institute (MNI) space using a high-level nonlinear warping algorithm, that is, the diffeomorphic anatomical registration through the exponentiated Lie algebra (DARTEL) technique (Ashburner 2007). Finally, each filtered functional volume was spatially normalized to the MNI space using the deformation parameters estimated during the above step and resampled into a 3 mm cubic voxel.

### Calculation of PCun functional gradients

**Fig. 2:**
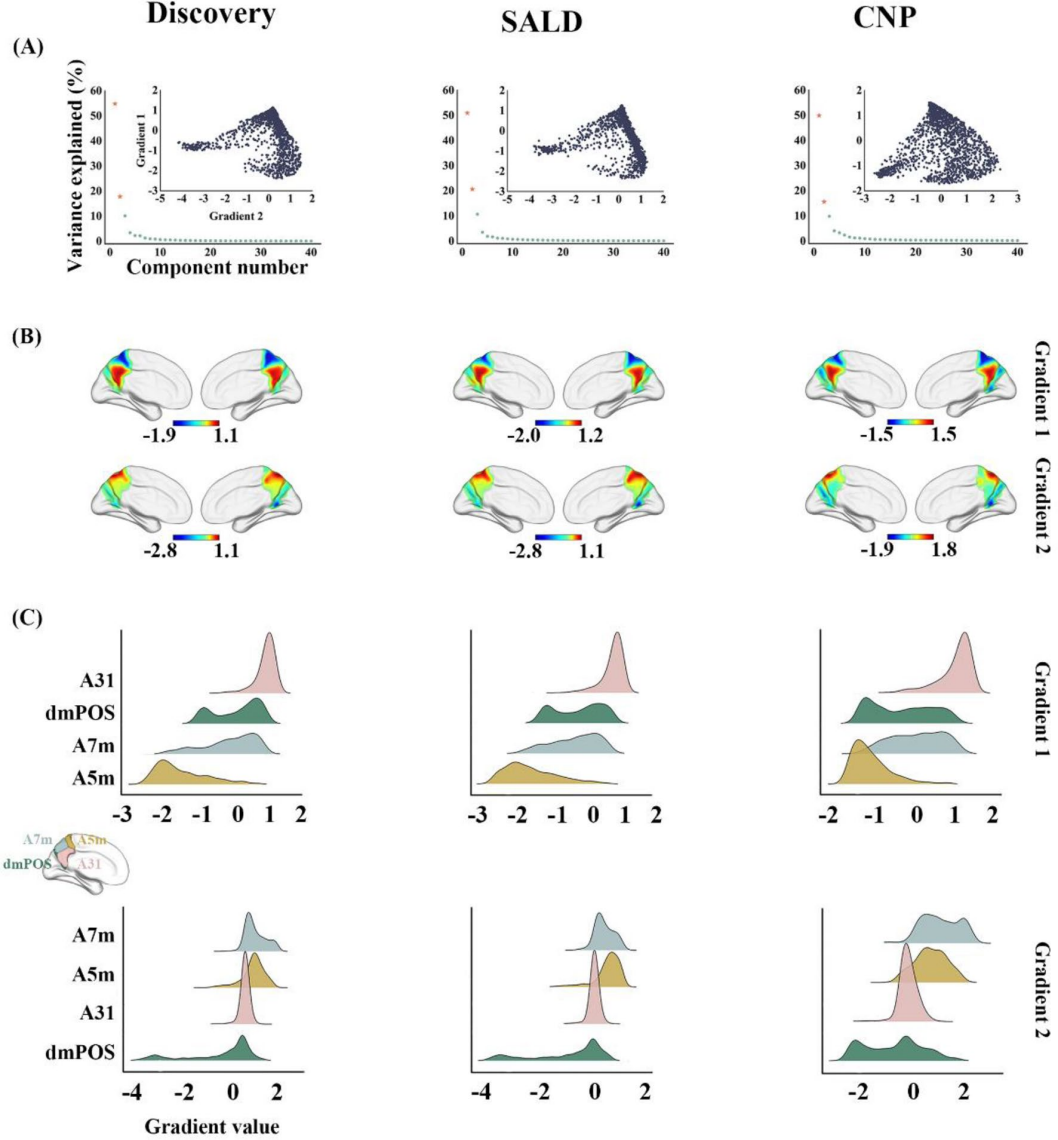
Functional gradients of the PCun in the discovery, SALD, and CNP datasets. **A** Variance explained by the functional gradients and inserted scatter plots of the first two gradients. **B** Topographies of the first two functional gradients. **C** Illustration of PCun subregions and their distributions along the first two gradients. Each histogram represents the distribution of gradient values of voxels within each PCun subregion. *PCun* precuneus; SALD, Southwest University Adult Lifespan Dataset, *CNP* Consortium for Neuropsychiatric Phenomics, *A7m* medial area 7, *A5m* medial area 5, *dmPOS* dorsomedial parietooccipital sulcus, *A31* area 31

Functional gradients of the PCun were calculated based on its rsFC to the entire cerebrum (Fig. 1). First, the Human Brainnetome Atlas (Fan et al. 2016), a new brain atlas constructed using a connectivity-based parcellation framework, was adopted to define the PCun (1,685 voxels) including medial area 7 (A7m), medial area 5 (A5m), dorsomedial parietooccipital sulcus (dmPOS), and area 31 (A31) in each hemisphere (Fig. 2C). Second, the preprocessed BOLD images were concatenated across all subjects after standardization using *z*-scores, yielding group-level BOLD time courses. Third, based on the group-level BOLD time courses, a voxel-wise PCun-to-cerebrum rsFC matrix (1,685 × 39,760) was generated by calculating Pearson’s correlation coefficients between time courses of each voxel within the PCun and each voxel within the cerebrum (excluding the PCun), followed by Fisher’s *Z*-transformation to improve normality. Then, for each row in the rsFC matrix, the values of the top 10% of connections were retained, whereas all others were zeroed (Margulies et al. 2016; Guell et al. 2018; Vos de Wael et al. 2018; Hong et al. 2019; Dong et al. 2020a). Fourth, similarity between all pairs of rows was calculated using cosine distance, resulting in a positive and symmetric affinity matrix representing similarity of connectivity profiles between each pair of voxels within the PCun.

We calculated PCun functional gradients using diffusion embedding (Coifman et al. 2005), a nonlinear dimensionality reduction technique that can recover a low-dimensional embedding from high-dimensional connectivity data. In the embedding space, voxels that are strongly connected by either many connections or few very strong connections are close, whereas voxels with little or no connections are far apart. Relative to other non-linear dimensionality reduction algorithms, diffusion embedding is relatively robust to noise, computationally inexpensive, and provides a stable representation of connections (Lafon and Lee 2006). By applying this algorithm to the affinity matrix, we identified multiple low-dimensional gradients explaining connectivity variance in descending order. For each gradient, a gradient value was assigned to each voxel within the PCun, resulting in a PCun gradient map to visualize macroscale continuous transitions in overall connectivity patterns, i.e., the gradient topography. We demonstrated the variance explained by first 40 gradients, and selected the first two gradients explaining the highest variations. Of note, the diffusion embedding is controlled by a single parameter α, which controls the influence of the density of sampling points on the underlying manifold (α = 0, maximal influence; α = 1, no influence). In line with previous studies (Margulies et al. 2016; Guell et al. 2018; Hong et al. 2019), we set α = 0.5 that is considered well-suited for the analysis of brain connectivity data.

### Relevance to gray matter volume

To determine the structural basis of PCun functional gradients, we examined their relationships with gray matter volume (GMV). Voxel-based morphology (VBM) approach was used to calculate GMV. First, all structural images were visually inspected to screen for artifacts or gross anatomical abnormalities; second, the structural images were segmented into gray matter, white matter and cerebrospinal fluid using the standard segmentation model; third, after initial affine registration into the MNI space, the gray matter concentration map was non-linearly warped using the DARTEL technique; finally, the GMV map was obtained by multiplying the gray matter concentration map by the non-linear determinants derived from the spatial normalization step. Then, cross-voxel Pearson’s correlation analyses were performed to examine the spatial associations between functional gradients and group-averaged GMV within the PCun. Nonparametric permutation tests were pursued to determine the statistical significance of the associations. Briefly, we adopted the brainSMASH toolbox (https://github.com/murraylab/brainsmash), based on the spatial-lag model (Burt et al. 2020), to generate 5000 surrogate PCun maps with spatial autocorrelation matched to that of the PCun gradient maps (i.e., 5000 permutations) and repeated the above-mentioned gradient-GMV correlations using the shuffled data. The gradient-GMV correlation coefficient in each permutation was recorded to build a null distribution. Based on the null distribution, the *P* value was calculated as the number of permutations that generated correlation coefficients greater than the true correlation coefficient/5000.

### Relevance to intrinsic geometry

To investigate whether PCun functional gradients were related to intrinsic geometry of the PCun, we calculated the Euclidean distance between the peak voxel of each gradient map and the remaining voxels within the PCun, resulting in a Euclidean distance map per gradient. Then, cross-voxel Pearson’s correlation coefficient between each PCun gradient map and the corresponding Euclidean distance map was calculated to index the extent to which each gradient changed with spatial distance from the maximal gradient location. The statistical significance of correlation was assessed using random permutation testing (5000 permutations). Notably, the correlations between PCun functional gradients and spatial distance were examined in each hemisphere, separately.

### Relevance to functional networks

To characterize the functional implications of PCun gradients, we evaluated their associations with canonical functional networks from the seven-network parcellation (Yeo et al. 2011). A PCun functional atlas was initially created with use of a custom winner-take-all parcellation method (Yang et al. 2020). That is, we calculated Pearson’s correlation coefficient between BOLD time course of a given voxel within the PCun and the average BOLD time course of each functional network. This PCun voxel was then assigned to the functional network with the highest Pearson’s correlation coefficient. This procedure was repeated for all voxels within the PCun, resulting in a PCun functional atlas including seven functional subdivisions corresponding to seven canonical functional networks. Finally, we extracted the gradient values of voxels within these functional subdivisions and sorted them by the median.

### Relevance to behavioral domains

To capture the behavioral relevance of PCun functional gradients, we investigated their associations with behavioral domains from the NeuroSynth (http://www.neurosynth.org), a well-validated and publicly available platform for large-scale automated synthesis of human neuroimaging data (Yarkoni et al. 2011). The NeuroSynth database provides a wide range of activation (*z*-statistics) maps of 1335 behavioral terms that describe conceptually distinct aspects of human behavior. To establish a link between gradient and behavior, each gradient map was binned into ten-percentile increments and then binarized, yielding 10 binary masks ranging from 0–10% to 90–100%. For each behavioral term, the average *z*-statistics within the 10 masks were extracted. The terms with *z*-statistic > 1.645 were used for visualization and interpretation.

### Sensitivity analyses

We performed several sensitivity analyses to verify the robustness of our results. First, before calculating the affinity matrix, we retained the top 10% of connections per row in the rsFC matrix. To assess the influence of threshold selections, our analysis was repeated with two other thresholds (top 20 and 30%) in discovery dataset. Second, to further exclude the potential influence of neurodevelopment and neurodegeneration, we repeated the functional gradient analyses in participants with a narrow age range of 18–30 years.

## Results

### Functional gradients of the PCun

The PCun functional gradient analyses showed consistent results across the discovery, SALD, and CNP datasets. Specifically, the variability in rsFC patterns of the PCun explained by the functional gradients is presented in descending order (Fig. 2A). The principal gradient (G1) accounted for the greatest variance in connectivity (discovery: 54.82%; SALD: 50.94%; CNP: 49.88%) and the secondary gradient (G2) explained the second-most variance (discovery: 17.79%; SALD: 20.63%; CNP: 15.60%). Scatter plots demonstrated the distributions of PCun G1 and G2. The topographies of G1 and G2 are presented in Fig. 2B. G1 showed a dorsoanterior-ventral organization, characterized by a gradual increase from the dorsoanterior portion (A5m) to the ventral portion (A31) of the PCun (Fig. 2C); G2 showed a ventroposterior-dorsal organization, manifested as a gradual increase from the ventroposterior portion (dmPOS) to the dorsal portion (A7m) of the PCun.

### Relevance to gray matter volume

**Fig. 3:**
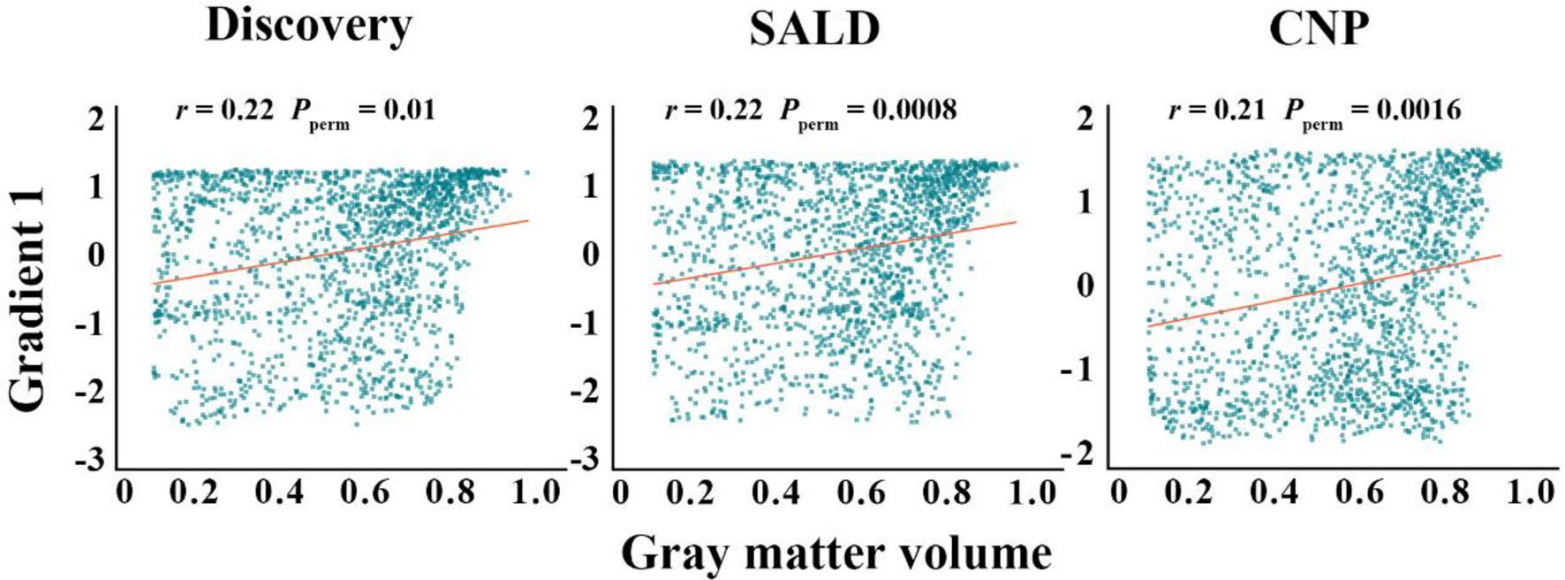
Scatter plots of the spatial correlations between PCun G1 and gray matter volume. *SALD* Southwest University Adult Lifespan Dataset, *CNP* Consortium for Neuropsychiatric Phenomics, *PCun* precuneus, *G1* gradient 1

Motivated by the hypothesis that brain function can be shaped and constrained by brain structure, we examined the associations between PCun functional gradients and GMV. Spatial correlation analyses revealed consistent positive associations between G1 and GMV across the three datasets (discovery: *r* = 0.22, *P*_perm_ = 0.01; SALD: *r* = 0.22, *P*_perm_ = 0.0008; CNP: *r* = 0.21, *P*_perm_ = 0.0016) (Fig. 3). However, the spatial correlations between G2 and GMV were not significant (discovery: *r* = 0.10, *P*_perm_ = 0.27; SALD: *r* = 0.13, *P*_perm_ = 0.12; CNP: *r* = 0.03, *P*_perm_ = 0.39). These results suggested that G1 was shaped, but not limited, by the underlying anatomy.

### Relevance to intrinsic geometry

**Fig. 4:**
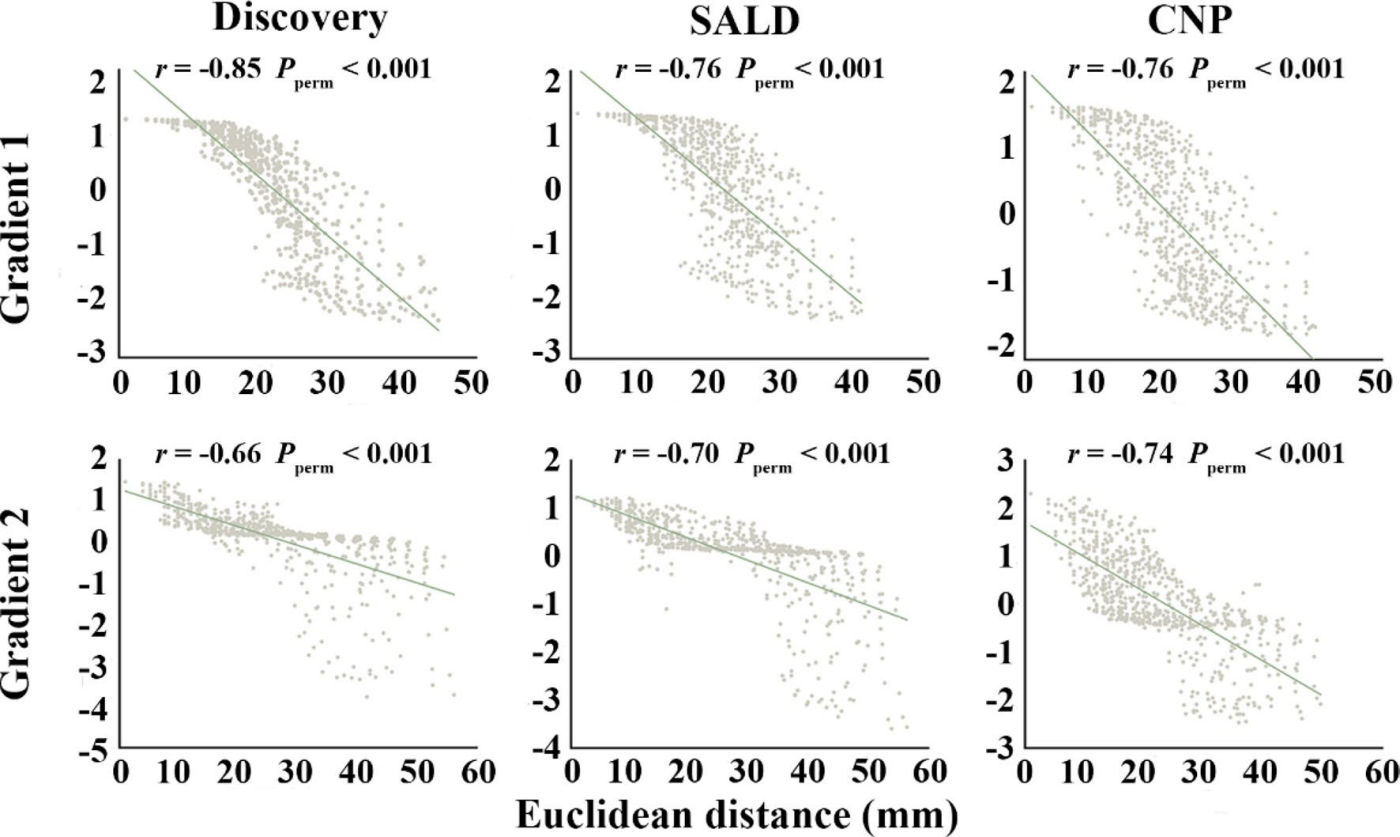
Scatter plots of the associations of G1 and G2 with spatial distance from the maximal gradient location in the left PCun. *SALD* Southwest University Adult Lifespan Dataset, *CNP* Consortium for Neuropsychiatric Phenomics, *PCun* precuneus, *G1* gradient 1, *G2* gradient 2

We examined the spatial associations between PCun functional gradients and the corresponding Euclidean distance maps to investigate their relationships with intrinsic geometry of the PCun. Cross-voxel Pearson’s correlation analyses demonstrated significant negative associations of G1 (discovery: *r* = − 0.85; SALD: *r* = − 0.76; CNP: *r* = − 0.76; *P*_perm_ < 0.001 for all) and G2 (discovery: *r* = − 0.66; SALD:* r* = − 0.70; CNP: *r* = − 0.74; *P*_perm_ < 0.001 for all) with spatial distance from the maximal gradient location in the left PCun (Fig. 4). This was also the case for the right PCun (Figure S1 in the Supplementary materials). These results suggested that G1 and G2 were related to intrinsic geometry of the PCun.

### Relevance to functional networks

**Fig. 5:**
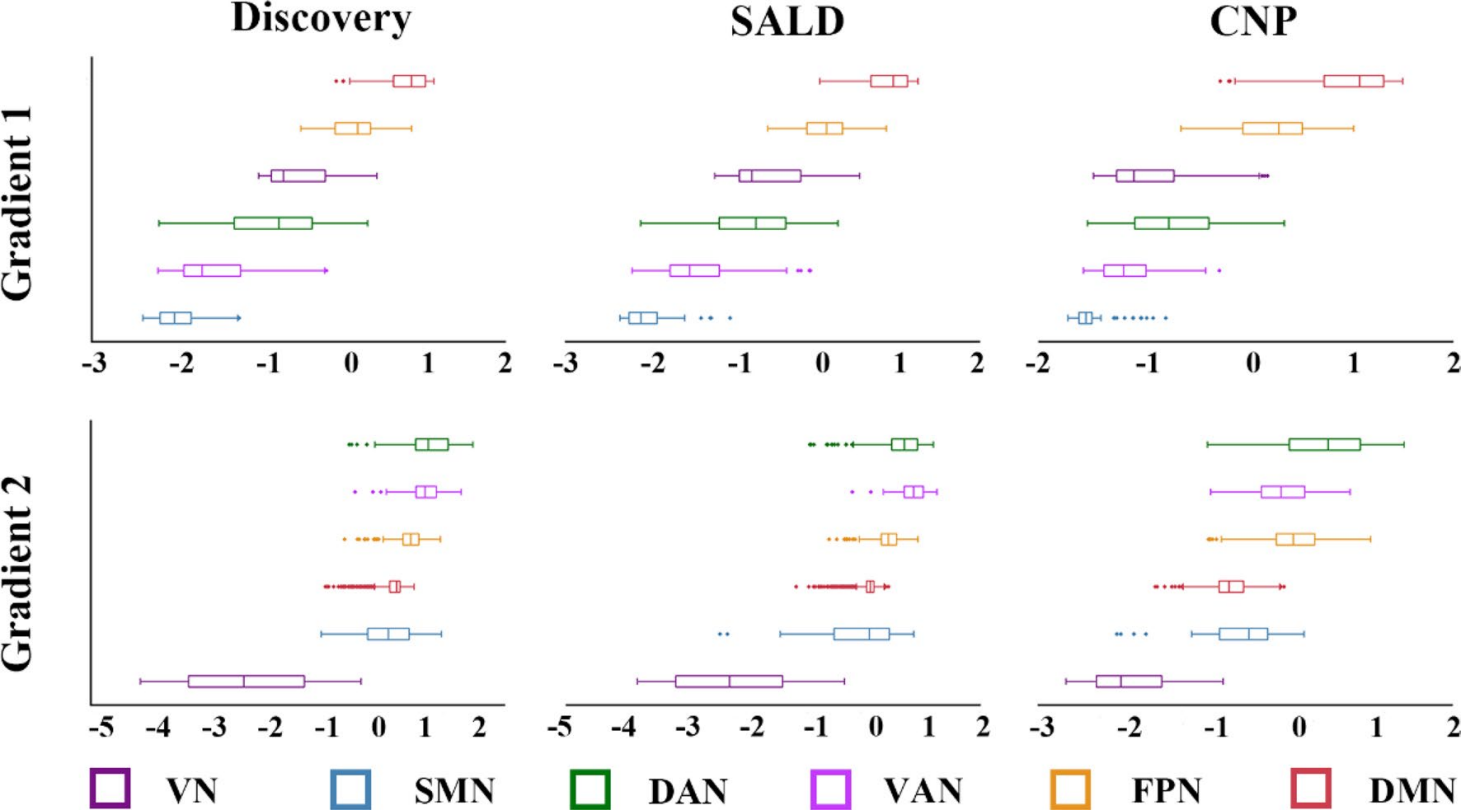
Box plots showing distributions of the PCun functional subdivisions corresponding to the canonical functional networks along G1 and G2. *VN* visual network, *DMN* default mode network, *SMN* sensorimotor network, *FPN* frontoparietal network, *DAN* dorsal attention network, *VAN* ventral attention network, *SALD* Southwest University Adult Lifespan Dataset, *CNP* Consortium for Neuropsychiatric Phenomics, *PCun* precuneus, *G1* gradient 1, *G2* gradient 2

The PCun functional subdivisions corresponding to the canonical functional networks were not randomly distributed along G1 and G2, but rather tended to cluster at similar positions (Fig. 5). Along G1, the functional subdivision corresponding to the sensorimotor network occupied one extreme position and was maximally separated from that corresponding to the default mode network at the other extreme; along G2, the functional subdivision corresponding to the visual network occupied one extreme position and was maximally separated from that corresponding to the dorsal attention network at the other extreme. Note that the functional subdivision corresponding to the limbic network was not found.

### Relevance to behavioral domains

**Fig. 6:**
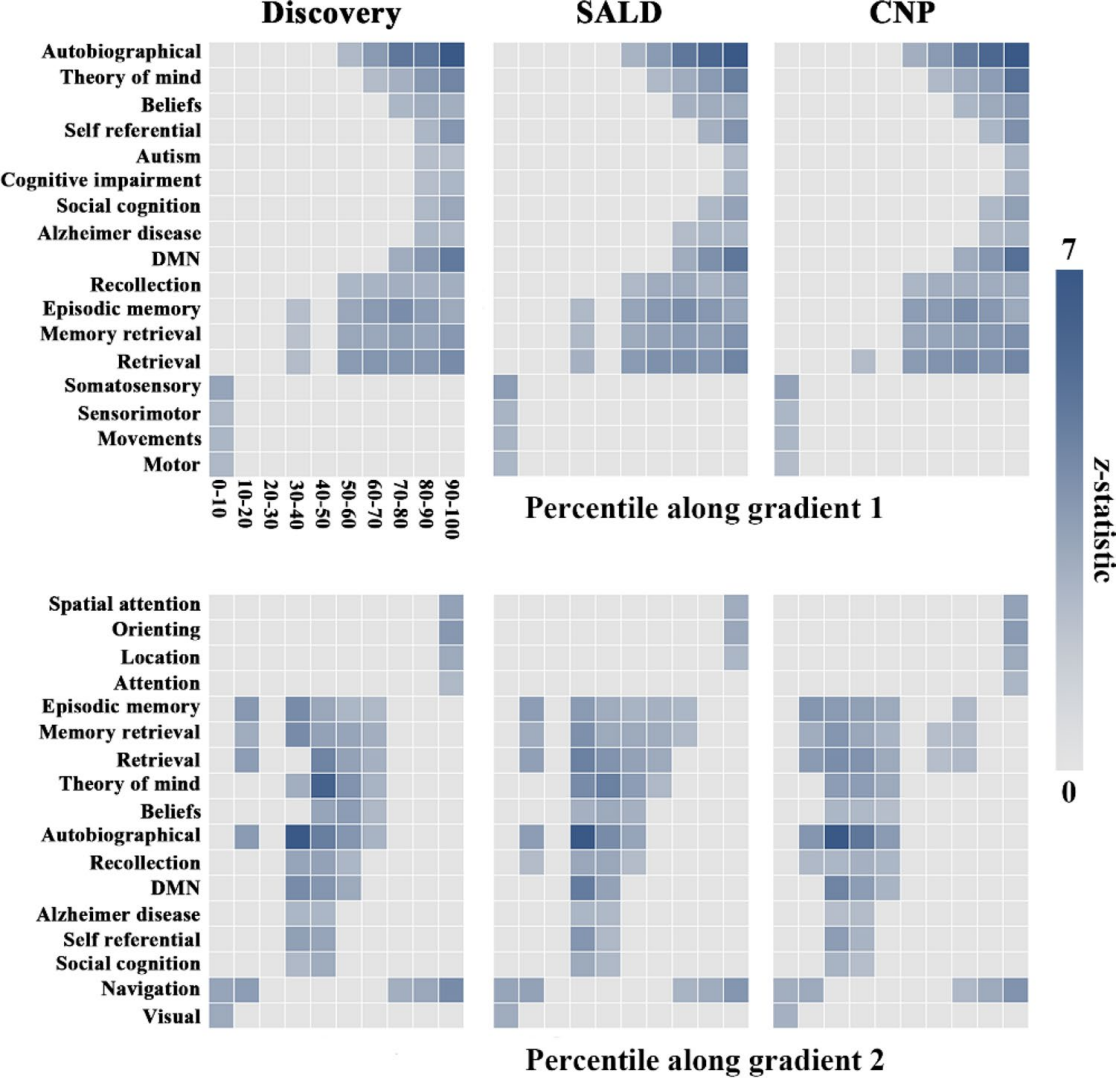
Associations of PCun G1 and G2 with behavioral terms from the NeuroSynth. To establish a link between gradient and behavior, each gradient map was binned into ten-percentile increments and then binarized, yielding 10 binary masks ranging from 0–10 to 90–100%. For each behavioral term, the average *z*-statistics within the 10 masks were extracted. The terms with *z*-statistic > 1.645 were used for visualization and interpretation, whereas those with *z*-statistic < 1.645 were zeroed. *SALD* Southwest University Adult Lifespan Dataset, *CNP* Consortium for Neuropsychiatric Phenomics, *PCun* precuneus, *G1* gradient 1, *G2* gradient 2

Behavioral relevance of PCun G1 and G2 was captured with use of the NeuroSynth. This analysis brought forward an important observation echoing the above-described results of functional network analysis. For G1, the end involving the sensorimotor network was linked to behavioral terms describing somatic movement and sensation such as “motor”, “movements”, “sensorimotor” and “somatosensory”, whereas the other end involving the default mode network was linked to terms describing abstract cognitive functions such as “autobiographical”, “theory of mind”, “beliefs” and “self referential”; for G2, the end implicating the visual network was related to terms depicting vision like “visual” and “navigation”, while the other end implicating the dorsal attention network was related to terms depicting top-down control of attention like “spatial attention”, “orienting”, “location” and “navigation” (Fig. 6).

### Sensitivity analyses

First, by applying two other thresholds (top 20% and 30%) to the rsFC matrix, we found that explained connectivity variance and topographies of the first two PCun functional gradients were highly consistent with those using the threshold of top 10% (Figure S2 in the Supplementary materials). Second, analyses in participants with a narrow age range of 18–30 years yielded G1 and G2 similar to those found in the whole sample (Figure S3 in the Supplementary materials). These results indicated that our findings were robust against these methodological variations.

## Discussion

By applying the state-of-the-art functional gradient approach to rs-fMRI data from discovery and validation samples, the present study opens new perspectives by being the first to systematically examine the hierarchical organization of the PCun. We found that PCun G1 showed a dorsoanterior-ventral organization from the A5m to the A31, and G2 exhibited a ventroposterior-dorsal organization from the dmPOS to the A7m. Concurrently, G1 was associated with cortical morphology, and both G1 and G2 showed geometric distance dependence. Importantly, PCun functional subdivisions corresponding to canonical functional networks (behavioral domains) were distributed along both gradients in a hierarchical manner, i.e., from the sensorimotor network (somatic movement and sensation) at one extreme to the default mode network (abstract cognitive functions) at the other extreme for G1 and from the visual network (vision) at one end to the dorsal attention network (top-down control of attention) at the other end for G2.

The high-dimensionality of brain features lies in the fact that more than one feature is typically assigned to each brain location. Examples are regional macro- and micro-structure, structural and functional connectivity, functional coactivation, gene or receptor expression, and particularly multimodal integrative features (Glasser et al. 2016; Eickhoff et al. 2018). In this instance, dimensionality reduction methods are needed to extract intelligible information from such high-dimensional data. One common approach is to group brain locations into larger parcels based on feature similarity (i.e., brain parcellation). However, treating parcels as discrete and independent entities may fail to capture more gradual changes and overarching spatial relationships (Jbabdi et al. 2013). Gradient approaches instead find the main axes of variance in the data through decomposition or embedding techniques, and replace the original high dimensions of brain features with a more parsimonious set of new dimensions (i.e., large-scale gradients) that explain most of the feature variance. Each new dimension is a continuous representation of one aspect of brain topographic organization, and each brain location can be described by a value reflecting where it falls along this dimension. There is now comprehensive evidence that the spatial arrangement of brain locations along these large-scale gradients is not arbitrary, but a consequence of developmental mechanisms shaped through evolutionary selection (Huntenburg et al. 2018). Studying the brain with respect to these large-scale gradients can inform our understanding of how the complex brain structure emerges and gives rise to its elaborate functions.

Employing a combination of the functional gradient method and rsFC data, we identified two PCun functional gradients, that is, a principal dorsoanterior-ventral axis from the A5m (sensorimotor network) to the A31 (default mode network) and a secondary ventroposterior-dorsal axis from the dmPOS (visual network) to the A7m (dorsal attention network). The parallel analysis with use of the NeuroSynth database further corroborated the results of functional network analysis by demonstrating a network-behavior correspondence. Indeed, our findings are largely consistent with several earlier neuroimaging studies that have parcellated the PCun into subregions based on their specific functional and anatomical connectivity patterns (Cavanna and Trimble 2006; Zhang et al. 2014; Margulies et al. 2009; Cauda et al. 2010). Specifically, the dorsoanterior portion (A5m) is functionally connected to the sensorimotor cortex, insular cortex, superior parietal lobule, fusiform gyrus and middle cingulate cortex, suggesting its involvement in the sensorimotor network; while the ventroposterior portion (dmPOS) exhibits strong connections with the cuneus, calcarine sulcus and lingual gyrus, implying a part of the visual network (Zhu et al. 2018). Based on transmitter receptor distribution characteristics, prior research has also documented that the rostral PCun (A5m) resembles the somatosensory cortex, whereas caudal PCun (dmPOS) is more similar to the visual cortex (Scheperjans et al. 2005). As a transition zone from the PCun to the posterior cingulate cortex (Cavanna 2007), the ventral portion (A31) shows great connectivity with the medial prefrontal cortex, anterior and posterior cingulate cortex, angular gyrus, lateral temporal cortex, indicating a core node of the default mode network. Cavanna and Trimble et al. (2006) reviewed functional imaging findings and demonstrated a prominent role of the ventral PCun in self-related processing and episodic memory retrieval. The dorsal portion (A7m) has widespread connections with the lateral prefrontal cortex, superior parietal lobule, angular gyrus, temporo-parietal junction area, and temporo-occipital junction area. These connections highlight the role of the dorsal PCun in cognitive/associative functions including attention (Zhang et al. 2014; Margulies et al. 2009). Collectively, our findings, in conjunction with prior reports, confirm the heterogeneous nature of the PCun. More importantly, the current work accommodates overlapped spatial distribution and continuous transitions of the PCun hierarchical organization, complementing and extending previous parcellation literature in an elegant way.

The relationship between brain structure and function is an important topic in systems neuroscience, which is crucial for understanding neurodevelopment, brain disorders, behavior and cognition (Paquola et al. 2019; Lariviere et al. 2020; Sporns et al. 2005; Vazquez-Rodriguez et al. 2019). Our data showed that PCun morphology was associated with G1 but not G2, indicating that the former appears to have a structural basis. This finding coincides with the traditional view that brain function is shaped, but not limited, by the underlying anatomy. In addition, we found that both G1 and G2 were linked to intrinsic geometry, with the lower gradient location being further away from the maximal gradient location. The current observation of geometric distance dependence is coherent with previous studies on functional gradients of the cerebral cortex, primary somatosensory cortex, and thalamus (Margulies et al. 2016; Ngo et al. 2021; Yang et al. 2020), suggesting a common feature of the topographic layouts mapped by functional gradients.

This study has several limitations. First, our analyses focused on the first two PCun functional gradients that explained the greater connectivity variance. However, some biologically relevant gradients with smaller explained variance might be overlooked. Second, to obtain more stable and reliable results, the PCun functional gradients were computed at the group level rather than at the individual level. Nevertheless, this may obscure meaningful individual variation. Finally, it is generally accepted that brain functional connectivity is shaped and constrained by structural connectivity (Honey et al. 2007). In future studies, diffusion MRI data will be collected to further investigate whether and how white matter structural connectivity may influence functional gradients of the PCun.

In conclusion, by applying the functional gradient approach to large-scale discovery and validation rs-fMRI datasets, we comprehensively characterized two hierarchical patterns of PCun topographic organization as well as their relationships with cortical morphology, intrinsic geometry, canonical functional networks, and behavioral domains. Our findings may provide mechanistic insights into the multifaceted nature of PCun heterogeneity. More broadly, prominent involvement of PCun abnormalities in many neuropsychiatric disorders highlights the potential of the PCun functional gradients to generate new hypotheses about disease mechanisms.

## Supplementary Information

Below is the link to the electronic supplementary material.

Supplementary file1 (DOC 1843 KB)

## Data Availability

In this study, we used brain imaging data from the Consortium for Neuropsychiatric Phenomics (CNP) (https://openneuro.org/datasets/ds000030/versions/1.0.0), and the Southwest University Adult Lifespan Dataset (SALD) (http://dx.doi.org/10.15387/fcp_indi.sald).
