# Supplementary material for: The hierarchical organization of the precuneus captured by functional gradients
Source: Brain Struct Funct. 2023 Jun 28;228(6):1561–72. doi: 10.1007/s00429-023-02672-5 (PMC10335959; doi:10.1007/s00429-023-02672-5)
Supplement: Supplementary file 1 — Supplementary file1 (DOC 1843 KB) [file 429_2023_2672_MOESM1_ESM.doc]

**Supplementary materials**

**Table S1. Demographic information of the discovery and validation datasets**

| **Dataset** | **Sample size** | **Age (years)** | **Gender (F/M)** | **FD (mm)** |
| --- | --- | --- | --- | --- |
| Discovery | 361 | 28.84 ± 10.83 | 183/178 | 0.13 ± 0.07 |
| SALD | 329 | 37.81 ± 13.79 | 207/122 | 0.15 ± 0.08 |
| CNP | 103 | 30.87 ± 8.56 | 47/56 | 0.17 ± 0.08 |

Age and FD are expressed as mean ± standard deviation. Abbreviations: SALD, Southwest University Adult Lifespan Dataset; CNP, Consortium for Neuropsychiatric Phenomics; F, female; M, male; FD, frame-wise displacement.

**Table S2.** Resting-state fMRI parameters for three datasets

| **Parameters** | **Discovery** | **SALD** | **CNP** |
| --- | --- | --- | --- |
| Scanner | 3.0T General Electric Discovery MR750w | 3.0T Siemens Trio | 3.0T Siemens Trio |
| Sequence | GRE-SS-EPI | GRE-EPI | T2^*^-weighted EPI |
| TR (ms) | 2000 | 2000 | 2000 |
| TE (ms) | 30 | 30 | 30 |
| FA (°) | 90 | 90 | 90 |
| FOV (mm^2^) | 220 × 220 | 220 × 220 | 192 × 192 |
| Matrix size | 64 × 64 | 64 × 64 | 64 × 64 |
| Slice thickness (mm) | 3 | 4 | 3 |
| Slice gap (mm) | 1 | 1 | - |
| Slices | 35 | 32 | 34 |
| Time points | 185 | 242 | 152 |

Abbreviations: fMRI, functional magnetic resonance imaging; SALD, Southwest University Adult Lifespan Dataset; CNP, Consortium for Neuropsychiatric Phenomics; GRE, gradient echo; SS, single shot; EPI, echo planar imaging; TR, repetition time; TE, echo time; FA, flip angle; FOV, field of view.


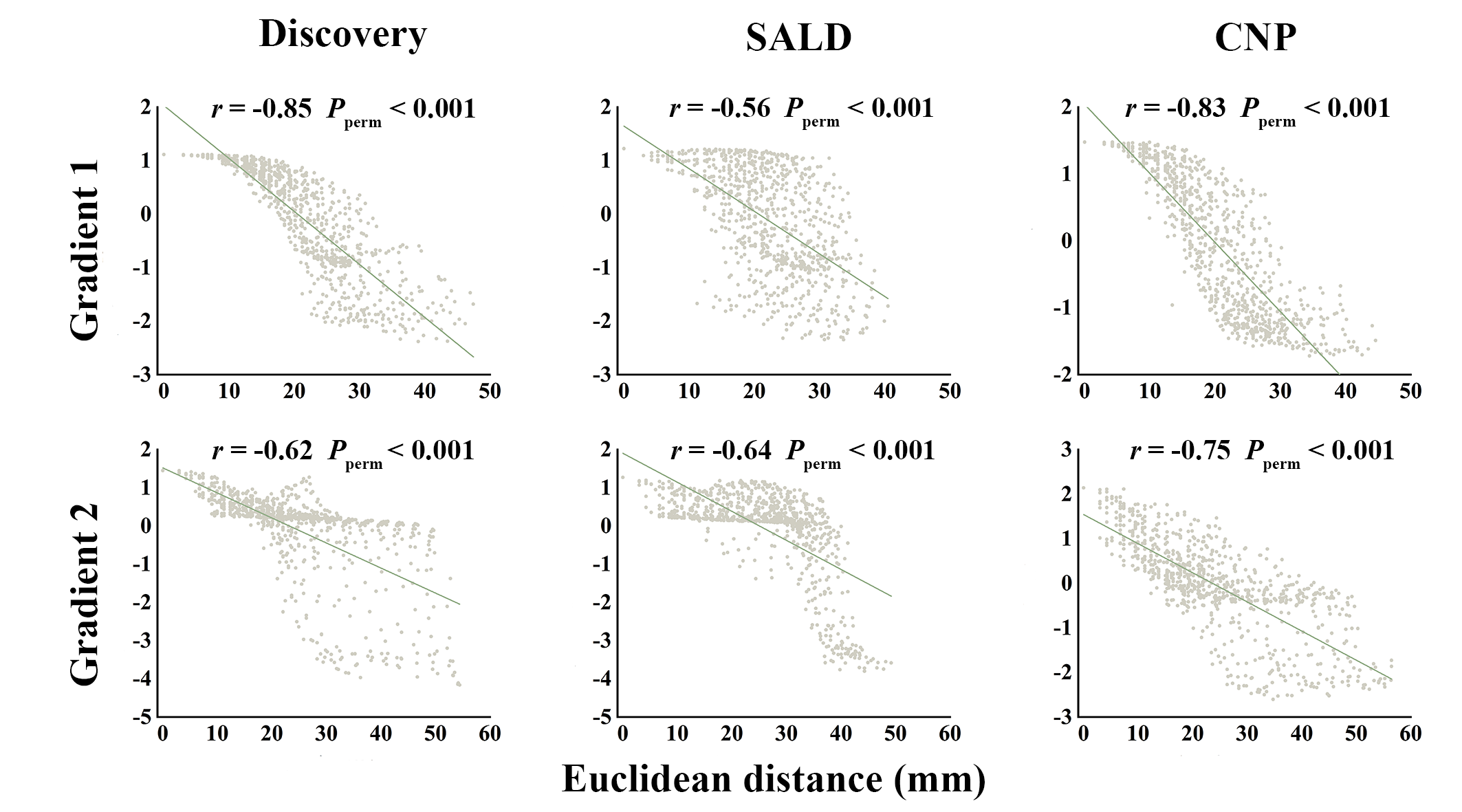


**Fig. S1** Scatter plots of the associations of G1 and G2 with spatial distance from the maximal gradient location in the right PCun. Abbreviations: SALD, Southwest University Adult Lifespan Dataset; CNP, Consortium for Neuropsychiatric Phenomics; PCun, precuneus; G1, gradient 1; G2, gradient 2.

**
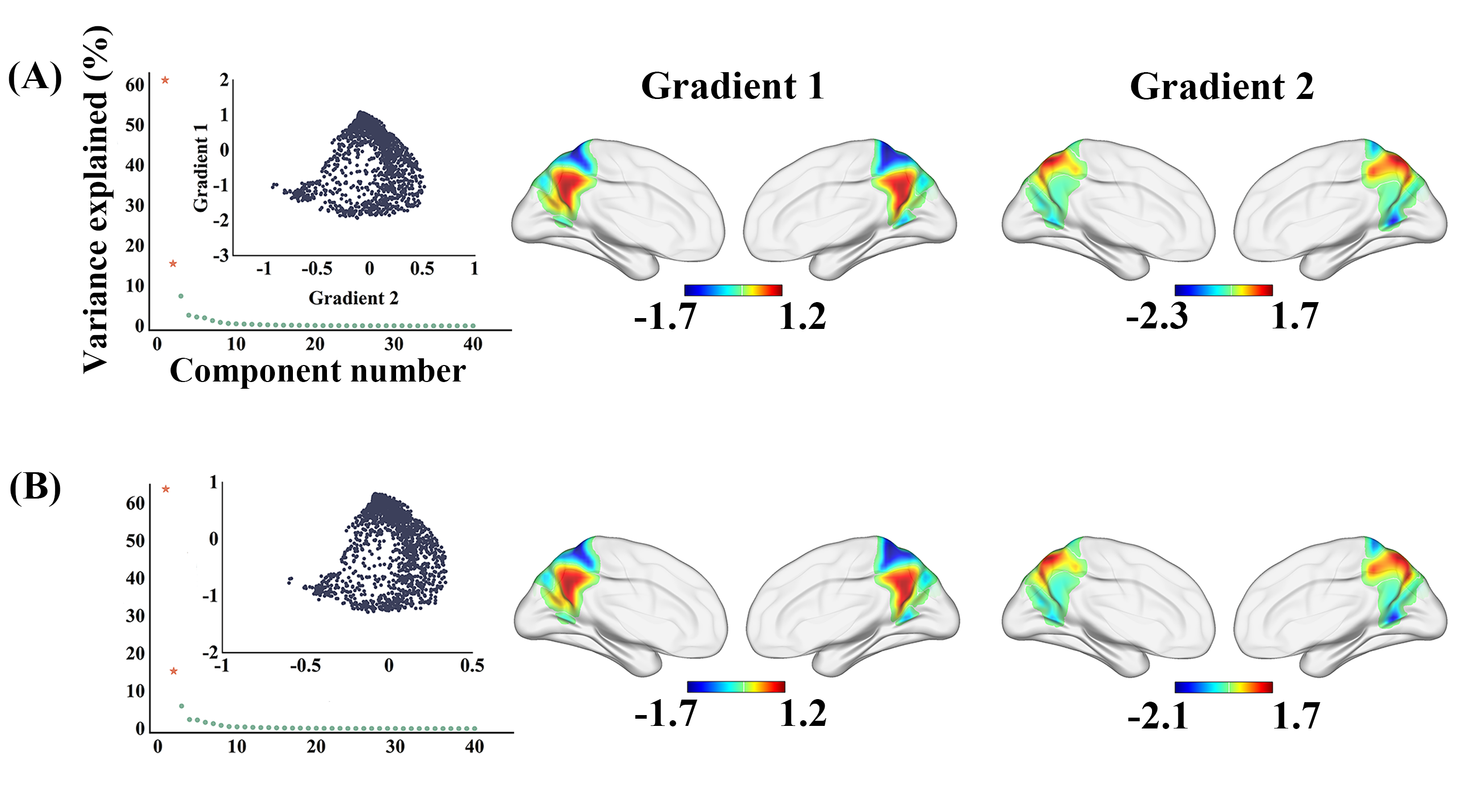
**

**Fig. S2** The first two PCun functional gradients derived from two other rsFC matrix thresholds of top 20% (A) and 30% (B) in the discovery dataset. On the left: connectivity variance explained by the functional gradients and inserted scatter plots of the first two gradients. On the right: topographies of the first two functional gradients. Abbreviations: PCun, precuneus; rsFC, resting-state functional connectivity.


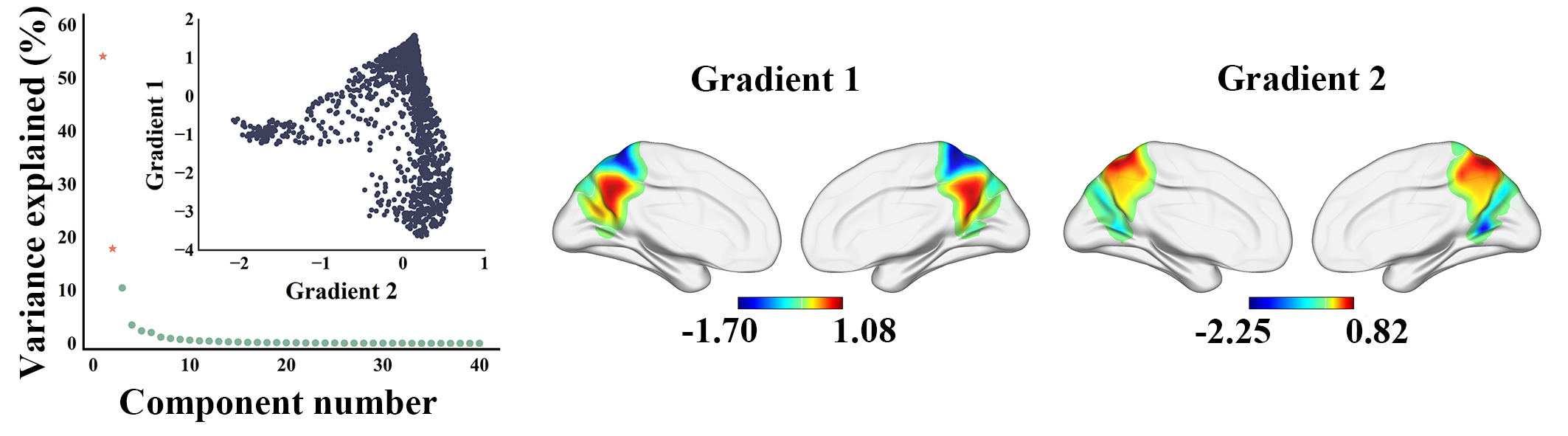


**Fig. S3** The first two PCun functional gradients calculated using participants with a narrow age range of 18-30 years. On the left: connectivity variance explained by the functional gradients and inserted scatter plot of the first two gradients. On the right: topographies of the first two functional gradients. Abbreviations: PCun, precuneus.
